# Comparison of Similarity Coefficients used for Cluster Analysis with Amplified Fragment Length Polymorphism Markers in the Silkworm, *Bombyx mori*

**DOI:** 10.1673/031.009.7101

**Published:** 2009-12-17

**Authors:** Seyed Benyamin Dalirsefat, Andréia da Silva Meyer, Seyed Ziyaeddin Mirhoseini

**Affiliations:** ^1^ Dept. of Sericulture, Faculty of Natural Resources, University of Guilan, Somehe Sara 1 144, Iran; ^2^ Uscola Superior de Agricultura “Luiz de Queiroz”, Departamento de Ciências Exatas, Piracicaba, SP, Brazil; ^3^ Dept. of Sericulture, Faculty of Natural Resources, Univ. of Guilan, Somae sara 1 144, Iran

**Keywords:** genetic divergence, correlation analysis, co-occurrence, Jaccard, Sorensen-Dice, Simple matching

## Abstract

Establishing accurate genetic similarity and dissimilarity between individuals is an essential and decisive point for clustering and analyzing inter and intra population diversity because different similarity and dissimilarity indices may yield contradictory outcomes. We assessed the variations caused by three commonly used similarity coefficients including Jaccard, Sorensen-Dice and Simple matching in the clustering and ordination of seven Iranian native silkworm, *Bombyx mori* L. (Lepidoptera: Bombycidae), strains analyzed by amplified fragment length polymorphism markers. Comparisons among the similarity coefficients were made using the Spearman correlation analysis, dendrogram evaluation (visual inspection and consensus fork index - *CI_C_*), projection efficiency in a two-dimensional space, and groups formed by the Tocher optimization procedure. The results demonstrated that for almost all methodologies, the Jaccard and Sorensen-Dice coefficients revealed extremely close results, because both of them exclude negative co-occurrences. Due to the fact that there is no guarantee that the DNA regions with negative cooccurrences between two strains are indeed identical, the use of coefficients such as Jaccard and Sorensen-Dice that do not include negative co-occurrences was imperative for closely related organisms.

## Introduction

One of approaches that is commonly used in studies of genetic diversity within and among populations or groups of individuals, and is applied with all types of markers and organisms, is based on comparisons of individual genotypes within and between populations. In such cases a genetic similarity (or dissimilarity) matrix constructed from all potential pairwise combinations of individuals is used to characterize population structure based on relative affinities of each individual to all other individuals tested. This approach requires suitable methods for evaluating similarity between individuals, and it is particularly useful in the case of possible linkages between different loci. The choice of an appropriate coefficient of similarity is a very important and decisive point to evaluate clustering, true genetic similarity between individuals, analyzing diversity within populations and studying relationship between populations, because different similarity coefficients may yield conflicting results (Kosman and Leonard 2005).

Silkworms, *Bombyx mon* L. (Lepidoptera: Bombycidae), domesticated for silk production, include a large number of geographical races and inbred lines that illustrate considerable differences in their qualitative and quantitative traits. Traits such as cocoon shape, cocoon color, silk fiber length, and ethological traits are used to differentiate silkworm varieties and selection of parental strains. But silkworm varieties, particularly those that have been bred from crosses involving many varieties, cannot be distinguished unambiguously by the use of conventional characteristics. It is thus apparent that the use of molecular markers could provide a solution to the problem by providing unique DNA profiles. Such varietal DNA profiles would be useful in producing reliable estimates of genetic diversity, for the selection of parents for the development of elite hybrids, and to protect silkworm breeder's rights (Mirhoseini 1998, 2002; Reddy et al. 1999; Nagaraju and Goldsmith 2002).

Molecular markers are commonly used to characterize genetic diversity within or between populations or groups of individuals because they typically detect high levels of polymorphism. Furthermore, RAPDs and AFLPs are efficient in allowing multiple loci to be analyzed for each individual in a single gel run. In analyzing banding patterns of molecular markers, the data typically are coded as (0,1)-vectors, 1 indicating the presence and 0 indicating the absence of a band at a specific position in the gel. With diploid organisms and codominant markers, such as allozymes, RFLPs or SSRs, the banding patterns may be translated to homozygous or heterozygous genotypes at each locus, and the allelic structure derived is utilized for comparison between individuals (Peakall et al. 1995; Smouse and Peakall 1999; Maguire et al. 2002). More often, however, the binary patterns obtained are used directly in comparisons of similarity of individuals (Kosman and Leonard 2005).

A number of coefficients have been proposed (Sokal and Sneath 1963; Sneath and Sokal 1973; Johnson and Wichern 1988). Similarity coefficients specific for dichotomic (binary) variables, especially co-occurrence measures, are suggested for divergency studies based on dominant molecular markers, such as RAPD (Duarte et al., 1999). These coefficients utilize several explanations of similarity or dissimilarities by entire comparisons, and their values show a discrepancy from 0 to 1 (Skroch et al., 1992). Despite the fact that various coefficients are available, published studies often do not state their preference for any one in particular. Since clustering and ordination results can be influenced by this choice (Gower and Legendre 1986; Jackson et al. 1989), these coefficients need to be better understood, in order that the most efficient once can be utilized.

In the present study, the alterations caused by three commonly used similarity coefficients on the subsequent clustering and ordination analyses of seven Iranian native *B. mon* strains analyzed by AFLP markers were evaluated.

## Materials and Methods

Three most commonly used similarity coefficients; the Simple matching, Jaccard and Sorensen-Dice coefficients (Table 1) were compared among seven Iranian native silkworm strains including Guilan Orange (Gu Or), Baghdadi (Ba), Harati White (Ha Wh), Harati Yellow (Ha Ye), Khorasan Lemon (Kh Le), Khorasan Orange (Kh Or) and Khorasan Pink (Kh Pi) which were sampled from Iran Sericulture Research Center (*ISRC* located in Rasht, Guilan province.

The AFLP marker was analyzed as described by Vos et al. (1995) with ten enzyme-primer combinations. Only polymorphic bands were used for the construction of the binary value matrix, representing the absence and presence of bands by 0 and 1, respectively. Each band was considered as a locus.

Genetic similarity estimates (*gs_ij_*) between each pair of individuals (*i,j*) were performed for three similarity coefficients (Table 1). Similarity analyses were done with the NTSYS-pc ver. 2.02 software (Rohlf 1998). Similarity coefficients were compared using the Spearman's correlation coefficient (Hollander 1973). Dendrograms were produced according to the unweighted pair-group mean arithmetic method (UPGMA) using NTSYS-pc software. The different dendrograms were then compared using visual inspection and the consensus fork index *CI_C_* (Rohlf 1982), in an analogous form to that used by Duarte et al. (1999). This *CI_C_* index provides a relative estimate of the dendrogram similarities and was calculated using NTSYS-pc software.

The establishment of the clusters was also studied by the Tocher optimization procedure (Rao 1952), using the Gene Program (Cruz 2001). The greatest value of the set of smaller distances involving each individual studied was considered the inter-group distance limit.

Finally, the cluster methodology proposed by Cruz and Viana (1994) was used, which consists of making the dissimilarity matrix projection into a two-dimensional space. The similarity coefficients were compared regarding the efficiency of this obtained projection.

To do this, the following was considered:

- Correlation between the original distances and the distances obtained by two-dimensional dispersion
- Degree of distortion (1 - α), given by:

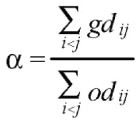

where *gd_ij_* is the graphical genetic distances between inbred lines *i* and *j*, in the two-dimensional space and *od_ij_* the original distances between lines *i* and *j,* in a *n*-dimensional space.
- Stress value (*S*), given by:

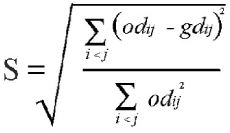

**Table 1.**
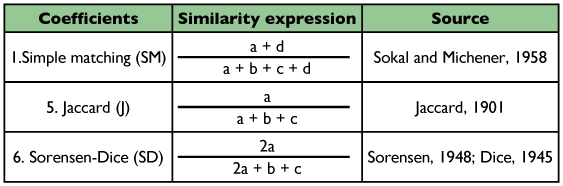
Similarity coefficients studied.

This statistical representation of stress (standardized residual sum of squares), proposed by Kruskal (1964), is a parameter that determines the goodness-of-fit of the graphic projection. The stress was classified according to the criteria presented in Table 2 (Kruskal 1964).

**Table 2.**
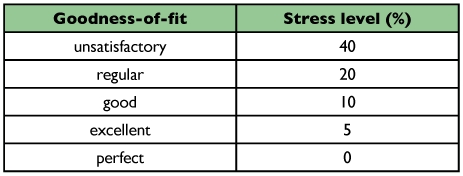
Stress (S) classification for the goodness-of-fit of the graphic projection.

## Results and Discussion

The Spearman correlation coefficients between the three similarity coefficients were equal to or close to 1 (Table 3), making it evident that they are highly related. The Jaccard and Sorensen- Dice coefficients presented correlation values equal to 1.00, demonstrating that there is no alteration in the ranks using any one of these coefficients, i.e. they classify the similarity among strains exactly in the same order. However, between these two classes of coefficients and the Simple matching coefficient, the correlations were lower (0.87). These results are similar to those presented by Duart et al. (1999) for RAPD markers in common bean and Meyer et al. (2004) for AFLP and RAPD markers in maize.

**Table 3.**
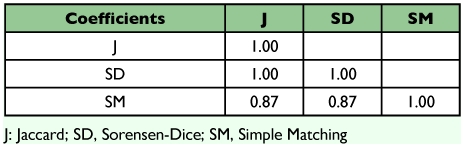
The Spearman correlation coefficient between the similarity coefficients

**Figure 1.**
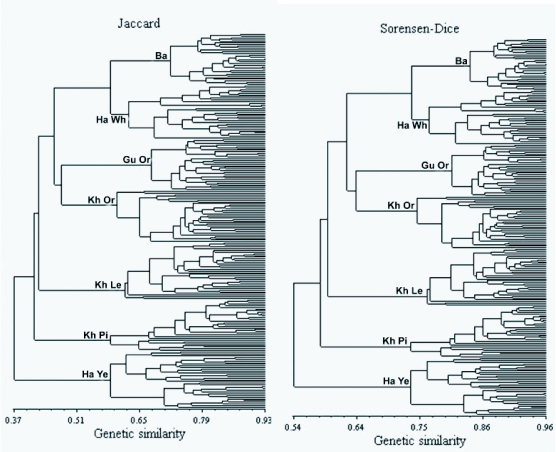
DDendrograms constructed for the seven Iranian native silkworm populations obtained from genetic similarities based on Jaccard, Sorensen-Dice and Simple matching similarity coefficients for the AFLP molecular markers (UPGMA) obtained from genetic similarities based on Jaccard, Sorensen-Dice and Simple matching similarity coefficients for the AFLP molecular markers (UPGMA). The strains were Guilan Orange (Gu Or), Baghdadi (Ba), Harati White (Ha Wh), Harati Yellow (Ha Ye), Khorasan Lemon (Kh Le), Khorasan Orange (Kh Or) and Khorasan Pink (Kh Pi)

A visual inspection of the dendrograms obtained with the UPGMA method (Figure 1) shows that, although the common structure of the dendrograms is highly comparable, there are minor alterations in the levels in which strains are clustered. The dendrogram constructed by Simple matching coefficient shows some distinct differences corroborating the similarity matrices outcomes (Table 3). Although the three coefficients made it possible to group four strains including Ba, Ha Wh, Gu Or and Kh Or in a main cluster, the Simple matching coefficient-based dendrogram, in the other hand, revealed some alterations in the grouping other strains including Ha Ye, Kh Le and Kh Pi (Figure 1). This also corroborates the differences observed in the similarity matrices (Table 3). It is important to note that, in the dendrogram constructed by Simple matching coefficient, the Kh Pi strain was distantly clustered from two other strains (Kh Or and Kh Le). This may be due to the fact that these three strains were collected from Khorasan province, a geographical region located in northern east of Iran, it is expected that they would be closely clustered in the dendrogram. This is observed in the dendrograms constructed by Jaccard and Sorensen- Dice's similarity coefficient confirming their validity in the Simple matching coefficient.

All the dendrograms were able to separate the individuals of the seven different strains without any overlapping that could be due to the high efficiency of the AFLP marker system used. It may also be a result of selection that has been carried out to conserve these strains, as well as the work carried out by the Iran Sericulture Research Center. In the past twenty years these native strains that have been collected from different geographical regions of Iran are inbreed by the Iran Sericulture Research Center to conserve the gene bank. The high similarity observed between individuals within each strain and consequent separation of strains can be due to the selection and inbreeding pressure. This result is in agreement with those obtained in the recent study using AFLP markers on *B. mori* (Mirhoseini et al. 2007). In contrast, earlier analysis using RAPD markers on individuals from the same strains (Mirhoseini 1998) did not lead to such consistent separation of the strains. The difference found here might be due to the different techniques used. The AFLP markers clearly resulted in a more consistent pattern.

The comparison of the constructed dendrograms by the consensus fork index *CIc,* allows a refinement of what is observed through visual inspection (Table 4). By this index, whose amplitude goes from 0 to 1, two dendrograms are considered identical when the calculated value equals one. As shown in table 4, based on the *CIc* index only, the dendrograms obtained by Jaccard and Sorensen-Dice's similarity coefficients are identical. These results are highly similar to those obtained by Duart et al. (1999) and Meyer et al. (2004). Highly coherent results were also obtained by Jackson et al. (1989), who studied relationships between different fish species based on different similarity coefficients and verified that cluster analysis shows a strong similarity between dendrograms obtained with Jaccard and Sorensen-Dice's coefficients, and Simple matching and Rogers and Tanimoto's coefficients.

**Table 4.**
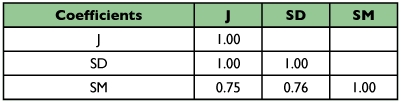
Consensus fork (CIc ) among the dendrograms (UPGMA) produced by similarity coeficients, based on Jaccard (J), Sorensen-Dice (SD) and Simple Matching (SM) similarity coeficients among seven Iranian native silkworm populations.

The similar appearance in Jaccard and Sorensen-Dice's coefficients-based dendrograms can be simplified by the properties of these coefficients. They are discriminated by the way in which the matrix of original data (1 = presence of the AFLP marker and 0 = absence) is employed in the similarity estimate. When two genotypes are compared, the following situations occur: *a* = 1.1; *b* = 1.0; *c* = 0.1; *d* = 0.0. Thus, Jaccard and Sorensen-Dice's coefficients are equivalent, except that double weight is given to positive co-occurrences (*a*) in the Sorensen-Dice's coefficient whereas the Simple matching coefficient includes negative co-occurrences (*d*) (Duart et al. 1999).

The Tocher optimization procedure (Rao 1952) is an individual clustering method that has been employed with dominant data *e.g.* RAPD and AFLP. In this method, individuals are separated into non-empty and equally exclusive sub-groups, based on the similarity or dissimilarity matrix (Cruz and Regazzi 1994), which can be obtained by several coefficients. However, it does not necessarily form the same groups as the dendrograms. Non-etheless, there is no information about the similarity of the strains inside each group or about similarity among the groups which can be considered a disadvantage of the method. In the present study, three coefficients revealed low alteration in the number of groups formed (See Supplementary Table) and also altered the classification of some strains in these groups. The results of this method agree with those observed by the dendrograms, considering the consensus fork index, i.e., confirming that the Jaccard and Sorensen-Dice coefficients are separated from the Simple matching coefficient. These results are highly constant with those earlier obtained by AFLP and RAPD markers (Duart et al. 1999 and Meyer et al. 2004).

The two-dimensional projection efficiency based on Kruskal (1964) classification (Table 2), revealed unsatisfactory stress values for all coefficients (Table 5). A similar result was obtained by Meyer et al. (2004), suggesting that this two-dimensional projection method is not adequate for this set of data, i.e., that the projections did not efficiently represent the similarity matrices. Therefore, the coefficients comparison under such conditions must be carefully made. Furthermore, the degree of distortion was high and the correlations were low under all conditions, confirming the latter. These results are different to those obtained by Duarte et al. (1999), in which the stress values varied from 11.4 to 32.0 (excluding the Russell and Rao coefficient). It was, therefore, possible to compare the efficiency of the coefficients.

**Figure:**
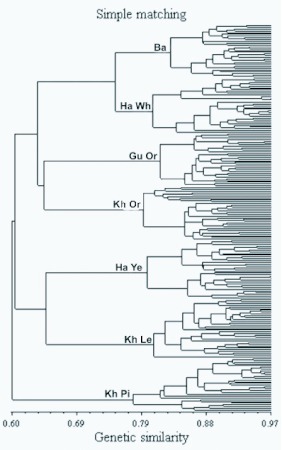

**Table 5.**
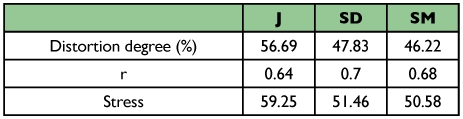
Distortion degree, correlation between the original and estimated distances (r) and stress value, obtained by the projection of the distances in the two-dimensional spaces.

Using the analyses described above that have distinct theoretical basis, some general tendencies were observed. The Jaccard and Sorensen- Dice coefficients can be separated from the Simple matching coefficient that always shows different results from the others. By inspection of their formulae, it can be perceived that the two first coefficients have common principles which differ from the third. The Jaccard and Sorensen-Dice coefficients do not consider the negative co-occurrences, while the Simple matching coefficient includes them in their expressions. This could possibly explain the different classification of the coefficients.

The Sorensen- Dice coefficient of similarity is frequently referred to as the measure of genetic similarity of Nei and Li (1979). For a given data set, the related values of Jaccard's similarity are always smaller than those of the Sorensen- Dice similarity and the simple matching coefficient. In contrast, values of the Sorensen- Dice similarity may be greater or smaller than the related values of the Simple matching coefficient depending on whether the number of positions with shared bands *a* is less or greater than the number of positions with shared absence of bands *d,* respectively.

The bases for choosing the most appropriate coefficient of similarity depend on type of marker and ploidy of the organism under consideration (Kosman and Leonard 2005). Landry and Lapoint (1996) suggested that the Sorensen- Dice or Jaccard coefficients might be preferable to the Simple matching coefficient when using RAPD analysis to compare groups of distantly related taxa. Hallden et al. (1994) considered the Simple matching coefficient to be the more appropriate measure of similarity when closely related taxa are considered, but Kosman and Leonard (2005) believe that choice should be supported with estimates of DNA sequence identity between the taxa. In the absence of supporting sequence identity estimates, similarity values based on dominant markers data should be regarded as tentative.

In their investigation, Kosman and Leonard (2005) could not recommend any preferred similarity measure for dominant markers in diploid (polyploid) organisms, because they believed that no suitable method could be proposed for measuring genetic similarity between diploid organisms on the basis of dominant banding profiles. In other words, banding patterns of diploids with dominant markers and polyploids with codominant markers represent individuals' phenotypes rather than genotypes. For the RAPD marker applied to common bean cultivars, Duarte et al. (1999) found greater efficiency in the two-dimensional projections for the Sorensen-Dice's coefficient, which was suggested for practical applications. However, we, and Meyer et al. (2004) did not find greater efficiency for this coefficient. As was stated by Meyer et al. (2004), based on the biochemical properties of the dominant markers, there is no guarantee that DNA regions with negative co-occurrences between two inbred lines are indeed identical. Consequently, it seems reasonable to conclude that the coefficients that exclude negative co-occurrences have more justification for being used when closely related organisms are being compared. Thus, it should be possible to use Jaccard or Sorensen-Dice to obtain satisfactory results when the organisms are closely related.

## Abbreviations

**AFLP**: amplified fragment length polymorphism,
**CIC**: consensus fork index,
**RAPD**: random amplification of polymorphic DNA,
**SSR**: simple sequence repeat,
**RFLP**: restriction fragment length polymorphism,
**Gu Or**: Guilan Orange,
**Ba**: Baghdadi,
**Ha Wh**: Harati White,
**Ha Ye**: Harati Yellow,
**Kh Le**: Khorasan Lemon,
**Kh Or**: Khorasan Orange,
**Kh Pi**: Khorasan Pink,
**UPGMA**: unweighted pair-group mean arithmetic method,
**NTSYSpc**: numerical taxonomy system
